# Identification of novel genetic variants, including PIM1 and LINC01491, with ICD-10 based diagnosis of pulmonary arterial hypertension in the UK Biobank cohort

**DOI:** 10.3389/fddsv.2023.1127736

**Published:** 2023-02-08

**Authors:** Alex Pu, Gautam Ramani, Yi-Ju Chen, James A. Perry, Charles C. Hong

**Affiliations:** Department of Medicine, University of Maryland School of Medicine, Baltimore, MD, United States

**Keywords:** pulmonary arterial hypertension, genome wide association study, PIM1, ICD-10, UK biobank

## Abstract

Pulmonary arterial hypertension (PAH) is characterized by remodeling and narrowing of the pulmonary vasculature which results in elevations of pulmonary arterial pressures. Here, we conducted a genome-wide association study (GWAS) using the UK Biobank, analyzing the genomes of 493 individuals diagnosed with primary pulmonary hypertension, based on ICD-10 coding, compared to 24,650 age, sex, and ancestry-matched controls in a 1:50 case-control design. Genetic variants were analyzed by Plink’s firth logistic regression and assessed for association with primary pulmonary hypertension. We identified three linked variants in the *PIM1* gene, which encodes a protooncogene that has been garnering interest as a potential therapeutic target for PAH, that were associated with PAH with genome wide significance, one (rs192449585) of which lies in the promoter region of the gene. We also identified 15 linked variants in the *LINC01491* gene. These results provide genetic evidence supporting the role of *PIM1* inhibitors as a potential therapeutic option for PAH.

## Introduction

Pulmonary hypertension (PH) describes a heterogeneous group of systemic, heart, and lung disorders characterized by elevations in pulmonary arterial pressures. Pulmonary arterial hypertension (PAH) represents a subgroup of PH, characterized by remodeling and narrowing of the pulmonary vasculature. Idiopathic pulmonary arterial hypertension (IPAH), which was previously coined primary pulmonary hypertension (PPH), describes PAH when no alternative etiology can be identified. Much of our understanding of the pathogenesis of PAH is shaped by the seminal human genetic studies which revealed that mutations in *BMPR2*, a gene encoding bone morphogenetic protein receptor type 2, account for ~80% of patients with hereditary PAH and ~20% of sporadic cases (Southgate et al., 2020; Ruopp and Cockrill, 2022). Subsequent exome sequencing and genome-wide association studies (GWAS) have found associations of PAH with additional genes, including those involved in the *SMAD* signaling pathway (Nasim et al., 2011) as well as in *KCNK3* (Ma et al., 2013), *SOX17* and *HLAD-DPA1/DPB1* (Gräf et al., 2018; Southgate et al., 2020). Nevertheless, there is an incomplete understanding of PAH pathogenesis and heterogeneity, including disease penetrance, onset, clinical course, and prognosis. Despite improved diagnosis and effective pharmacotherapy, PAH mortality remains high, ~10% per year (Southgate et al., 2020), highlighting a large unmet medical need.

Using the UK Biobank cohort, we have previously carried out ICD-10 (International Classification of Diseases 10)-based GWAS for a variety of cardiovascular diseases (Ashvetiya et al., 2021; Alkhalfan et al., 2022; Gyftopoulos et al., 2022). Here, using the UK Biobank cohort, we carried out a GWAS of loci associated with the ICD-10 diagnostic code for primary pulmonary hypertension (I27.0), which encompasses IPAH and hereditary PAH but not other forms of associated PAH, and found novel genetic variants, including those in the *PIM1* and *LINC01491* genes, associated with it.

## Methods

### Ethical approval

The present study, which involved deidentified data obtained from the UK Biobank Resource under Application Number 49852, received the proper ethical oversight, including the determination by the University of Maryland, Baltimore Institutional Review Board that the study is not human research (IRB #: HF-00088022).

### Study population

This study utilized data from the UK Biobank, a large ongoing prospective cohort study involving 506,682 participants between 2006 and 2010. Participants were aged 40–69 at the time of recruitment. Our study population was identified using the ICD-10 diagnostic code for primary pulmonary hypertension (I27.0) as either the participant’s main diagnosis (indicating reason for hospital admission) or secondary diagnosis code. This ICD-10 diagnostic code encompasses (1) IPAH, (2) hereditary PAH, (3) primary PAH, and (4) primary group 1 PH and excludes PAH due to secondary causes. This I27.0 code is most similar, but not identical, to Group 1 pulmonary arterial hypertension (PAH) based on the newer World Health Organization (WHO) grouping (Simonneau et al., 2019). Given the ambiguity of the definitions of “primary pulmonary hypertension,” we have henceforth classified each case as “pulmonary arterial hypertension (PAH).”

### Genome-wide association study

The UK Biobank contains genetic data with over 820,000 genotyped SNPs and up to 90 million imputed variants available. Using data from the UK Biobank Resource on 487,310 subjects with imputed genotypes, we performed quality control by removing those with genetic relatedness exclusions (Data-Field 22018—UKB, https://biobank.ctsu.ox.ac.uk/crystal/field.cgi?id=22018; 1532 subjects), sex chromosome aneuploidy (Data-Field 22019 –UKB, https://biobank.ctsu.ox.ac.uk/crystal/field.cgi?id=22019; 651 subjects), mismatch between self-reported sex and genetically determined sex (Data-Field 31 –UKB, https://biobank.ctsu.ox.ac.uk/crystal/field.cgi?id=31; Data-Field 22001 –UKB, https://biobank.ctsu.ox.ac.uk/crystal/field.cgi?id=22001; 372 subjects), recommended genomic analysis exclusions (Data-Field 22010—UKB, https://biobank.ctsu.ox.ac.uk/crystal/field.cgi?id=22010; 480 subjects), and outliers for heterozygosity or missing rate (Data-Field 22017 –UKB, https://biobank.ctsu.ox.ac.uk/crystal/field.cgi?id=22077; 968 subjects).

Additionally, we chose to limit subject selection to the UK Biobank’s genetic ethnic grouping described as “White British” with very similar genetic ancestry based on principal components analysis and made available as part of the UK Biobank Resource (Data-Field 2,206—UKB, https://biobank.ctsu.ox.ac.uk/crystal/field.cgi?id=22006).

We defined cases with the ICD-10 code I27.0 as specified above. The set of cases was purged of relatedness by removing one of each related pair in an iterative fashion until no related subjects remained. Relatedness was defined as a kinship value of 0.0442 or greater in the data provided from the UK Biobank Resource. For each identified case, 50 controls were selected from the remaining pool of unrelated participants, matching for age, sex, and ancestry. This was done with incremental tolerance expansion for age and ancestry. For age, the tolerance ranged from 0 (exact match) to a max of 7 years. For ancestry, principal components (PCs) supplied by the UK Biobank were used. We assessed the similarity in ancestry by calculating the mathematical distance in a graph plotting the PC1 × PC2. The tolerance for ancestry ranged from a calculated distance of 2 PC units to a maximum of 80 PC units, with PC1 ranging from 0 to +400 and PC2 ranging from −300 to +100.

To perform this GWAS, Plink’s firth logistic regression was used, adjusting for age, sex, and 5 PCs using data supplied by the UK Biobank Resource. Firth regression was used for analysis as it has been shown to yield the best combination of control for type I error and power for detecting low-frequency variants (Wang, 2014; Chang et al., 2015). The PAH cases were analyzed with the 40 million imputed genetic variants provided by the UK Biobank with imputation quality scores greater than 0.80. This analysis incorporated covariates of sex, age, and principal components 1 through 5 to adjust for ancestry. Precalculated PC data for the first 40 principal components were supplied by the UK Biobank. Only the first 5 PCs had significance with p-values <0.05 in our preliminary analysis. As a result, we only included the first 5 PCs for our analysis.

### Identification of pulmonary arterial hypertension variants

A minor allele frequency (MAF) of at least 0.5% and a significance value of < 5E-08 was used to identify variants of interest.

### Comorbidity and biomarker analysis

The UK Biobank also contains participant baseline clinical information, including comorbidities, height, weight, body mass index, and basic laboratory values. The ICD-10 code diagnoses, ABO blood type, 5 biometric markers, and 61 biomarkers in the identified PAH cases and the matched controls were examined. The significance of these conditions and variables with PAH patients in comparison to age, sex, and ancestry-matched controls were determined by two-sample *t*-test.

## Results

A total of 493 (243 males, 250 females) individuals were identified to have a diagnosis of PAH from a total of 472,124 participants, representing a prevalence of 1 in 1,000. Among these individuals, the mean (SD) age of diagnosis was 63.4 (8.21) years. Of the 490 cases with available BMI data, 355 (72%) had a BMI of 25 and above.

### Genome-wide association study

**FIGURE 1:**
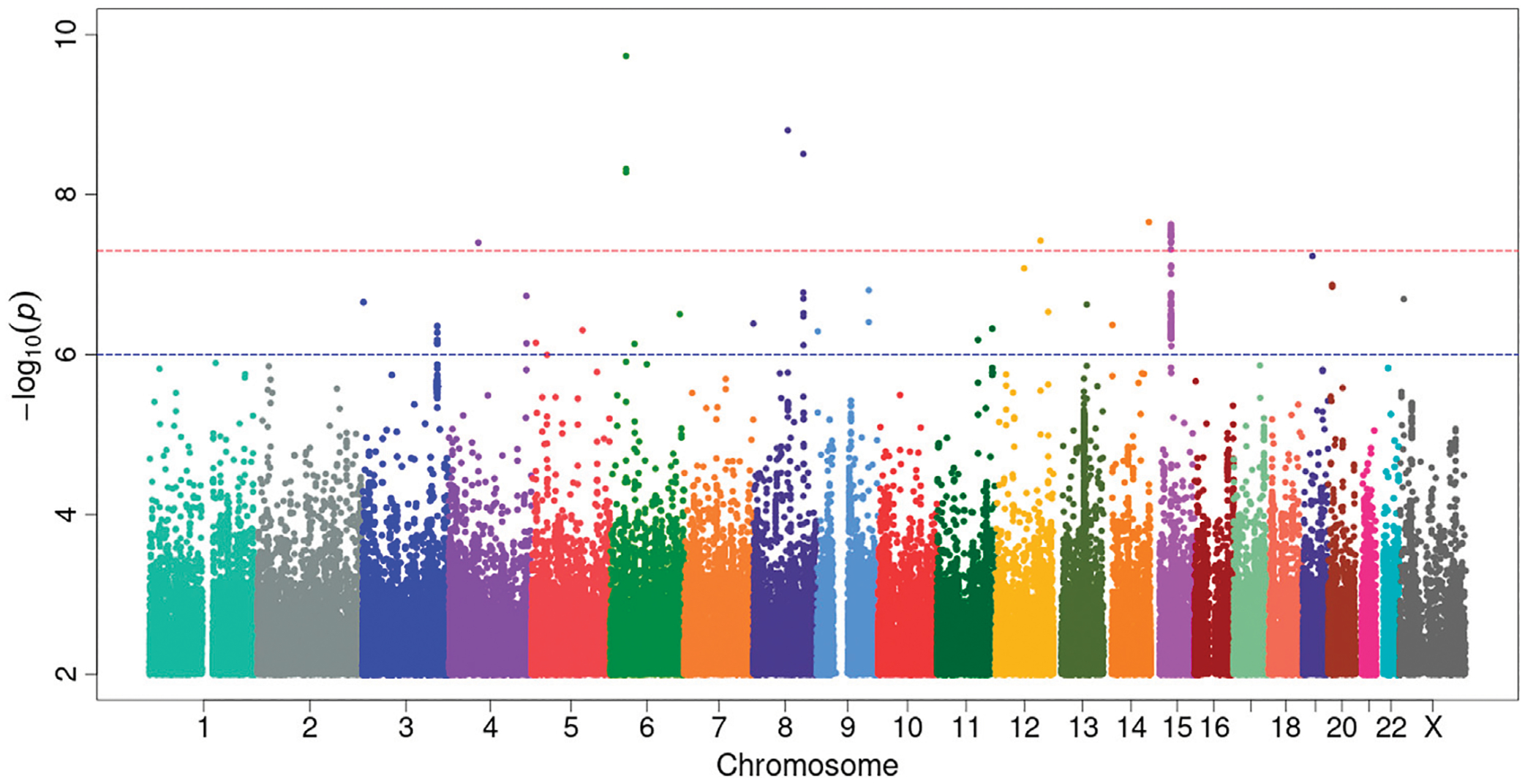
Genome-wide association study analysis for variants associated with pulmonary arterial hypertension. Manhattan plot of GWAS results with minor allele frequency >0.5% for PAH. The red dotted line denotes genome wide significance at *p* = 5E-8. The significance is displayed on the *y*-axis as -log10 of the *p*-value, and the results lie according to their chromosomal location on the *x*-axis.

**FIGURE 2:**
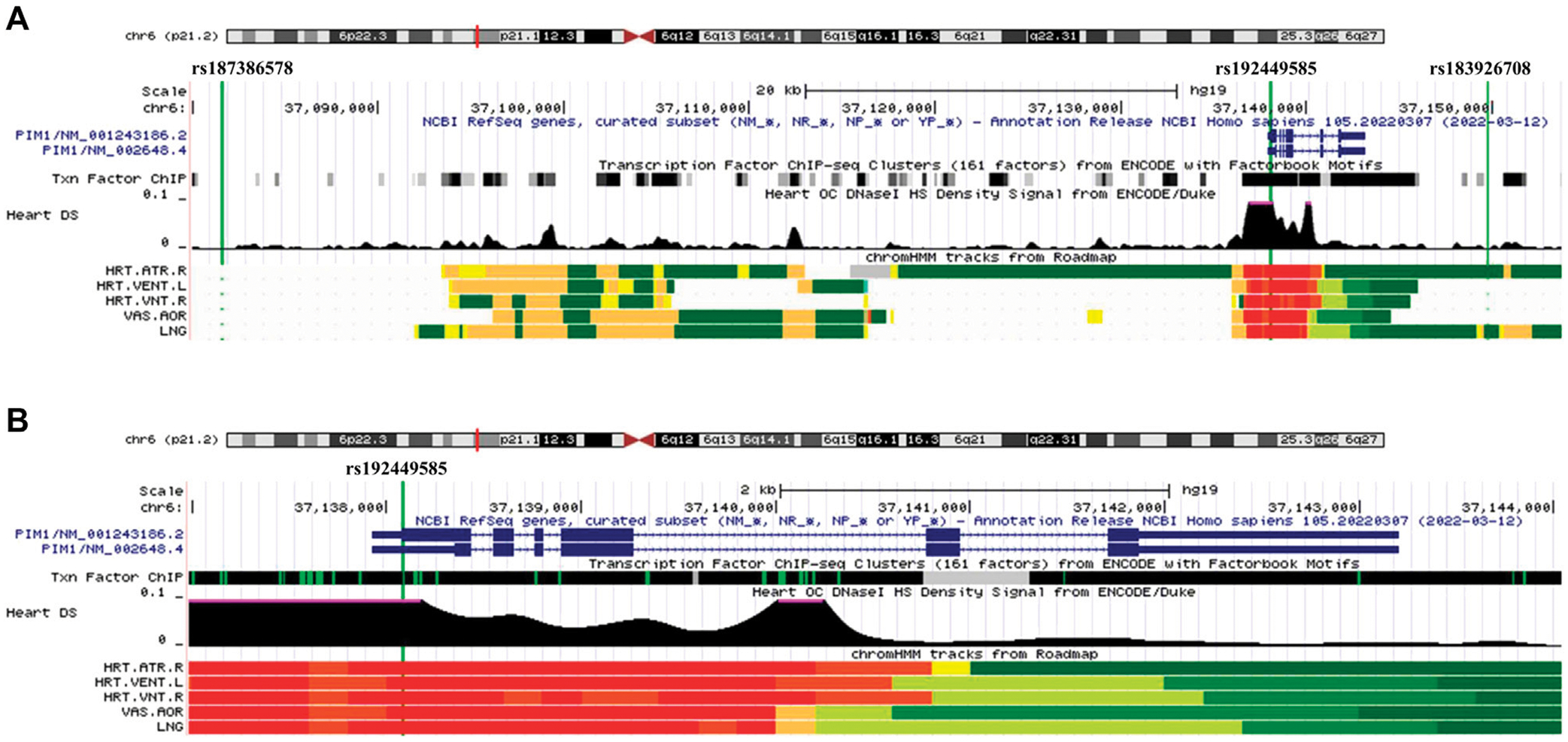
UCSC Genome Browser view of genomic region for *PIM1* variants. (**A**) UCSC Genome Browser for all three variants. Green vertical line depicts location of variant, labeled on top. Dark blue regions indicate the 6 exons for *PIM1* plus untranslated regions (5′ and 3′ UTRs are narrower dark blue sections). Black region of “Txn Factor ChIP” indicates density of transcription factor ChIP-seq clusters from ENCODE. Black graphic for “Heart DS” indicates the Dnase I hypersensitivity density signal from ENCODE/Duke. Bottom five tracks indicate the predicted chromatin state from Roadmap. Red indicates a transcription start site region for five tissues, HRT.ATR.R (Right Atrium), HTR.VENT.L (Left Ventricle), HRT.VNT.R (Right Ventricle), VAS.AOR (Aorta), LNG (Lung). (**B**) UCSC Genome Browser focused on exonic variant rs192449585 and *PIM1* gene.

**FIGURE 3:**
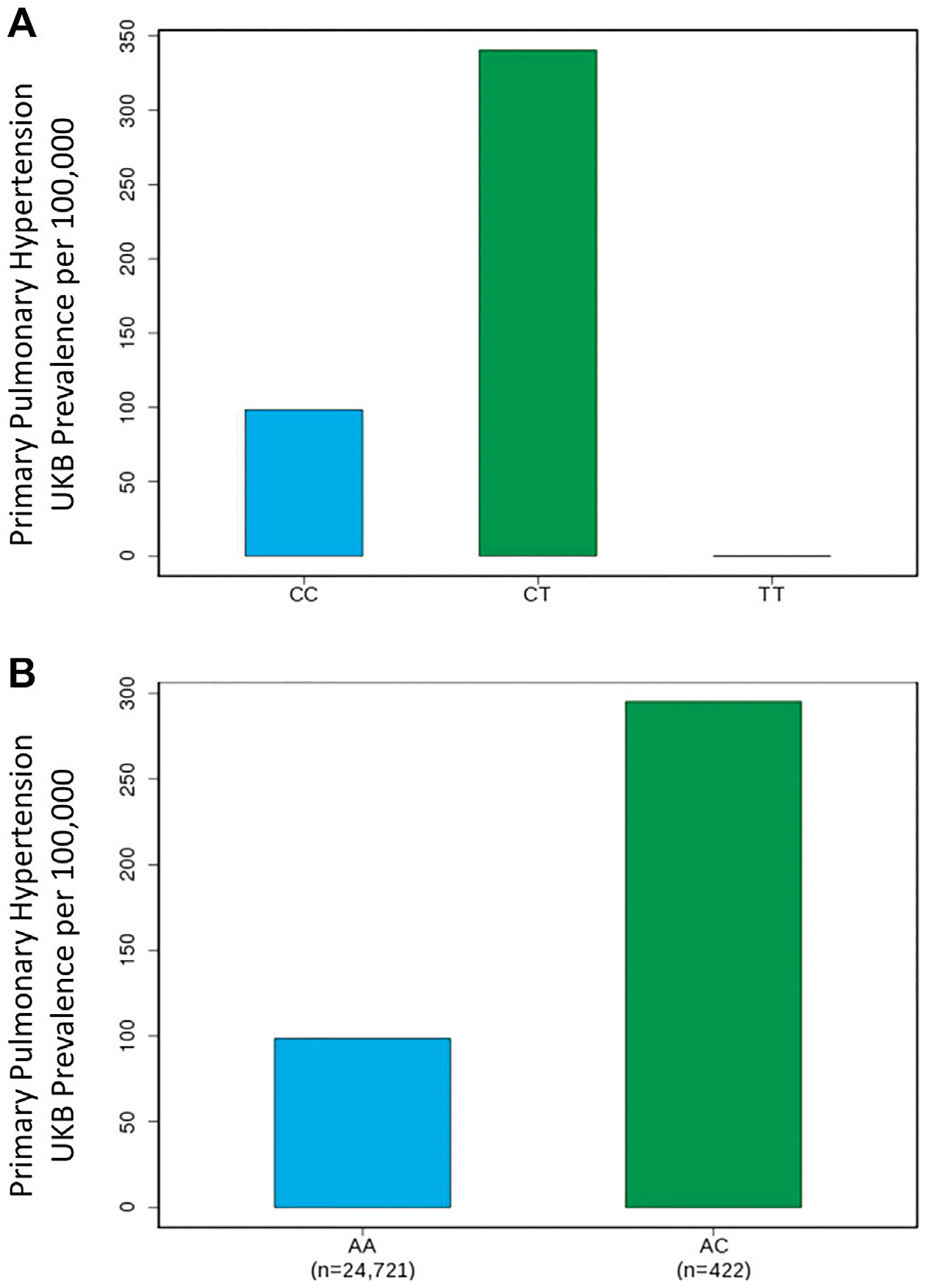
Genome-wide association study results for *PIM1* and *LINC01491* variants associated with pulmonary arterial hypertension. (**A**) Prevalence of PAH in the UK Biobank increases with *PIM1* variant rs192449585 status. CC denotes wildtype, CT denotes heterozygous carrier, TT denotes homozygous carrier. (**B**) Prevalence of PAH in the UK Biobank increases with *LINC01491* varian rs145733648 status. AA denotes wildtype, AC denotes heterozygous carrier.

Our GWAS of variants with MAF of at least 0.5% identified a total of 24 variants in 7 distinct loci (Figure 1). We identified 3 variants associated with PAH related to the *PIM1* gene, which encodes the Pim-1serine/threonine-protein kinase, which is involved in cytokine signaling, cell cycle progression and oncogenesis (Holder and Abdulkadir, 2014). One variant was rs192449585 (MAF 0.76%; OR 3.808; *p* = 4.78E-09), located in the first amino acid of the exonic region of the *PIM1* isoform 1 gene in a region of high density Dnase I hypersensitivity signals and predicted transcription start site for several tissues (Figure 2). Further, this variant lies in the transcription factor binding region of 28 transcription factors, including *POLR2A*, *IRF1*, *CTCR*, *TAF1*, and *TBP* (Figure 2, Supplementary Figure S1), and alters 13 regulatory motifs, including *TATA*, *YY1*, and *SIN3A* (Supplementary Table S1). Additionally, this variant was found to have an eigenPC score of 5.046, which are functional prediction scores ranging from −3.2 to +72 based on conservation, allele frequencies, deleteriousness prediction and epigenomic signals using an unsupervised learning method (Ionita-Laza et al., 2016). An eigenPC score of 5.046 places rs192449585 at the top 99.66th percentile of all variants. The prevalence of PAH in heterozygotes with the rs192449585 variant was 340 per 100,000, compared to 98 per 100,000 in the wild types (Figure 3A). Interestingly, two individuals were found to be homozygous for rs192449585, both of whom are not diagnosed with PAH and are females 49 years of age and older. The second variant was rs183926708 (MAF 0.75%; OR 4.002; *p* = 1.85E-10), located in the intergenic region −6,549 bases from the *PIM1* gene. The third variant was rs187386578 (MAF 0.75%; OR 3.626; *p* = 5.28E-09), located in the intergenic region 56,310 bases from the *PIM1* gene. The three variants were found to be linked in our linkage disequilibrium analysis. A locus zoom plot of rs183926708, the top variant, can be found in Supplementary Figure S2.

Our GWAS identified 15 variants related to *LINC01491*. All of the identified variants lie in the intergenic region of *LINC01491*. The top variant within this locus was rs145733648 (MAF 0.85%; OR 3.303; *p* = 2.34E-0.8). The prevalence of PAH in heterozygotes with the rs145733648 variant was 295 per 100,000, compared to 98 per 100,000 in the wild types (Figure 3B). Information regarding the other *LINC01491* variants can be found in Table 1. A locus zoom plot of rs145733648 can be found in Supplementary Figure S3.

In addition, 6 other variants at 5 other loci with MAF over 0.5% were found to be associated with PAH. Information regarding these can be found in Table 1, with information regarding the further breakdown of cases and controls for each variant in Supplementary Table S2. Of note, variant rs147444776 was identified in the intronic region of *FBN1* gene, which encodes fibrillin-1, a structural protein involved in elastic and non-elastic connective tissue. Quantile-quantile plots (QQ Plots) are provided in Supplementary Figure S4 to illustrate that the GWAS quality was well controlled.

### Comorbidities and biomarkers

The cases of PAH were more likely to be diagnosed with dyspnea (R06.0; 24% vs. 2%, *p* < 0.0001), chest pain (R07.4; 25% vs. 7%, *p* < 0.0001), syncope and collapse (R55; 14% vs. 3%, *p* < 0.0001), all of which are common symptoms of PAH. PAH cases were also more likely to be diagnosed with congestive heart failure (I500; 29% vs. 1%, *p* < 0.0001), chronic kidney disease (N18.9; 15% vs. 1%, *p* < 0.0001), and atrial fibrillation/flutter (I48; 53% vs. 6%, *p* < 0.0001). Additionally, PAH cases were more likely to report a family history of heart disease (Z82.4; 20% vs. 4%, *p* < 0.0001) than controls.

Interestingly, individuals with a diagnosis of PAH were also more likely to be diagnosed with left ventricular failure (I50.1; 25% vs. 1%, *p* < 0.0001), chronic obstructive pulmonary disease (J44.9; 20% vs. 3%, *p* < 0.0001), and pulmonary embolism (I26.9; 12% vs. 1%, *p* < 0.0001). Additionally, PAH cases were also more likely to have a co-diagnosis of hypothyroidism (E03.9; 13% vs. 5%, *p* < 0.0001), iron deficiency anemia (D50.9; 10% vs. 2%, *p* < 0.0001), sleep apnea (G47.3; 5% vs. 1%, *p* < 0.0001), non-insulin-dependent diabetes mellitus (E11.9; 21% vs. 6%, *p* < 0.0001), pure hypercholesterolemia (E78.0; 38% vs. 13%, *p* < 0.0001), and essential hypertension (I10; 65% vs. 29%, *p* < 0.0001). A full list of co-diagnoses can be found in Table 2.

On average, PAH cases had higher BMIs (28.68 vs. 27.51, *p* < 0.0001) than controls. Individuals with PAH had a higher mean serum urea (39.3 vs. 33.9 mg/dL, *p* < 0.0001), creatinine (0.824 vs. 0.735 mg/dL, *p* = 0.0001), uric acid (5.93 vs. 5.32 mg/dL, *p* < 0.0001) and cystatin C (1.11 vs. 0.939 mg/dL, *p* < 0.0001) levels, consistent with the higher rate of diagnosis of chronic kidney disease in that population when compared to controls. While individuals with PAH were more likely to be diagnosed with hypercholesterolemia, they had a lower mean cholesterol (201 vs. 221 mg/dL, *p* < 0.0001) compared to the control population. Consistent with findings regarding diabetes mellitus type 2, the PAH cohort had a higher HbA1c (5.7% vs. 5.5%, *p* < 0.0001) and fasting glucose level (97.3 vs. 93.3 mg/dL, *p* = 0.01) when compared to the control cohort. These results are shown in Table 3.

## Discussion

In our GWAS using data available from the UK Biobank, we discovered 3 linked variants in the *PIM1* gene associated with PAH. One of these variants (rs192449585) is particularly noteworthy because, while it does not alter the resulting amino acid sequence, it lies in the promoter region of the gene and overlaps with the binding sites of transcription factors such as RNA Polymerase as well as regulatory motifs such as TATA, suggesting it may impact *PIM1* expression. This is supported by the variant’s high eigenPC score of 5.046, an aggregate predictive score for functional importance, which helps to prioritize likely causal variants in a given genomic region (Jeffcott and Whitwell, 1973).

While *PIM1* is more commonly viewed as a key player in the role of tumorigenesis, Paulin and colleagues have provided evidence for its potential role in PAH (Paulin et al., 2011; Renard et al., 2013; Lampron et al., 2020). *PIM1* has been hypothesized to amplify the growth signals from *STAT3*, an isoform of the signal transducers and activators of transcription (STAT) protein family, to activate transcription factor nuclear factor of activated T cells (NFAT), which in turn promotes the pro-proliferative and anti-apoptotic phenotype of PAH (Paulin et al., 2011). While *STAT3* and *NFAT1* are constitutively expressed in normal pulmonary arteries, *PIM1* was found to only be present in PAH, making it a therapeutic target of interest. Further studies have corroborated this, showing that inhibition of *PIM1* leads to decreased *KU70* (lupus Ku autoantigen protein p70) recruitment to damaged DNA sites, effectively inhibiting DNA non-homologous end-joining, which allows for increased apoptosis in PAH pulmonary artery smooth muscle cells. In the same study, they found *PIM1* inhibition resulted in improved vascular remodeling and pulmonary hemodynamics in mice models (Lampron et al., 2020). To our knowledge, our study is the first to show a human genetic association of *PIM1* with PAH. Since pipeline drug targets with human genetic evidence of disease association are more likely to lead to approved drugs than those without such evidence (Nelson et al., 2015; Hong, 2022), our study lends important support for *PIM1* inhibition as a promising therapeutic approach for PAH. Moreover, as plasma *PIM1* levels have been associated with PAH disease severity, our findings further corroborate the role of *PIM1* in the pathogenesis of PAH and as a potential therapeutic target (Renard et al., 2013; Zhu et al., 2019).

In addition to our findings regarding *PIM1*, we also identified 15 linked variants in *LINC01491*, which encodes long intergenic noncoding RNA (lncRNA) 1491. While *LINC01491* has not been well characterized in the literature, other lncRNAs have been found to play a role in the pathogenesis of PAH (Han et al., 2021), with lncRNA H19 being a specific example showing promise as a biomarker for severity of PAH as well as a potential therapeutic target (Omura et al., 2020). Studies to verify our findings regarding *LINC01491* may be warranted to confirm its role in PAH pathogenesis and explore its utility as a biomarker or therapeutic target.

Our study also provides information corroborating our current understanding of PAH and associated comorbidities. For example, recent studies have found that iron deficiency is correlated with PAH disease severity, with a prevalence ranging from 30% to 65% in patients with IPAH (Rosenkranz et al., 2020). We found that our PAH cases were more likely to be diagnosed with iron deficiency and had slightly lower levels of hemoglobin compared to matched controls, although our findings showed a smaller prevalence of iron deficiency. Additionally, our study found that urine microalbumin levels were higher in PAH (Table 2), in comparison to controls, consistent with recent findings that low grade albuminuria is prevalent in PAH patients (Nickel et al., 2019). Moreover, we observed a higher prevalence of non-insulin-dependent diabetes and higher mean hemoglobin A1c in our PAH cases, consistent with prior studies linking insulin resistance to PAH (Zamanian et al., 2009; Pugh et al., 2011; Hemnes et al., 2019). Interestingly, our study found that patients with PAH generally had lower levels of Vitamin D and were more likely to have comorbid hypothyroidism. While the mechanism behind these findings is still unclear, our results are consistent with findings from prior epidemiological studies and further suggest that studies to elucidate these relationships may be warranted (Callejo et al., 2020; Rosenkranz et al., 2020).

There are several limitations in this study. First, the identification of genetic variants in the GWAS does not prove functional causality, and further studies should be done to confirm these findings. Additionally, these findings should be validated in a patient population with a more diverse genetic origin. The use of ICD-10 codes to select cases is also a limitation as many diseases may be misdiagnosed in the real world. In particular, PAH is often misdiagnosed, which is one reason referral to specialty centers is recommended. ICD-10 based studies offer an opportunity to study a wide array of diseases and medical conditions, especially rare ones like PAH, in a large, “real world” setting. The prevalence of PAH in the UK Biobank is much higher than the previously reported prevalence of 10–15 cases per million people (Ruopp and Cockrill, 2022), reflecting issues with the diagnosis of “primary” PH in the real world. This is further corroborated by the fact that, even though PAH by definition is a diagnosis that excludes left heart disease, pulmonary disease, and thromboembolic disease, there was a higher rate of co-diagnosis of these three conditions in our PAH cases. Additionally, the ICD-10 diagnosis code for “primary pulmonary hypertension” does not provide information about rare subtypes of Group 1 PH, including pulmonary veno-occlusive disease/pulmonary capillary hemangiomatosis or hereditary hemorrhagic telangiectasia associated PAH.

Our study also found markedly higher rates of chronic and acute renal failure, atherosclerotic heart disease, essential hypertension, and peripheral vascular disease as well as higher mean CRP levels in PAH patients. Given that these co-morbid conditions can be considered vascular diseases, one wonders whether PAH involves a more general vascular pathology. Regardless of these limitations and confounding factors, our findings identify novel genetic variants associated with PAH and provide further support for the notion of repositioning *PIM1* inhibitors like TP-3654, which was originally developed for advanced solid tumors (Lampron et al., 2020), for the treatment of PAH, possibly in conjunction with *PIM1* detection in pulmonary vasculature or elevated plasma *PIM1* levels.

## Conclusion

Using ICD-10 diagnostic codes for PAH, we identified 3 linked variants in the *PIM1* gene in our GWAS of the UK Biobank, further suggesting the role of *PIM1* in the pathogenesis of PAH in humans and providing key human genetic evidence in support of the potential of *PIM1* inhibitors as a therapeutic option for PAH. We also identified 15 linked variants in the *LINC01491* gene, contributing to the growing literature of lncRNAs contributing to the pathogenesis of PAH.

## Supplementary Material

Data Sheet 1

Data Sheet 3

Data Sheet 2

## Figures and Tables

**TABLE 1:** Detailed genome wide association study results.

rs number	Chr	Position	INFO	Type	Gene	OR (95% CI)	*p*-value	MAF (%)	eigenPC
rs567757955	4	68163311	0.875	intergenic	LOC101927237	3.88 (2.39,6.29)	3.97E-08	0.58	−0.215
rs183926708	6	37149753	0.867	intergenic	PIM1	4.00 (2.61,6.13)	1.85E-10	0.75	
rs192449585	6	37138079	0.828	exonic	PIM1	3.81 (2.43,5.96)	4.78E-09	0.76	5.046
rs187386578	6	37081612	0.903	intergenic	PIM1	3.63 (2.35,5.59)	5.28E-09	0.75	−0.21
rs370775256	8	79449318	0.888	ncRNA_intronic	LOC101927003	3.12 (2.16,4.51)	1.57E-09	1.23	
rs573886591	8	115176106	0.926	intergenic	CSMD3	3.20 (2.18,4.70)	3.10E-09	1.07	−0.229
rs4764961	12	103829793	0.806	intronic	C12orf42	2.11 (1.62,2.75)	3.76E-08	4.00	−0.222
rs193148583	14	104828824	0.883	intergenic	KIF26A	3.81 (2.38,6.09)	2.20E-08	0.65	0.211
rs145733648	15	48197300	0.981	intergenic	LINC01491	3.30 (2.17,5.02)	2.34E-08	0.85	−0.184
rs60777293	15	48182605	0.966	intergenic	LINC01491	3.30 (2.17,5.02)	2.51E-08	0.85	−0.023
rs113318990	15	48205457	0.968	intergenic	LINC01491	3.30 (2.17,5.03)	2.69E-08	0.86	0.059
rs80073095	15	48151703	0.928	intergenic	LINC01491	3.36 (2.19,5.16)	2.82E-08	0.83	−0.221
rs77487976	15	48202855	0.978	intergenic	LINC01491	3.26 (2.14,4.95)	3.06E-08	0.87	−0.125
rs61518006	15	48195875	0.969	intergenic	LINC01491	3.26 (2.15,4.95)	3.14E-08	0.87	−0.135
rs113347288	15	48194582	0.968	intergenic	LINC01491	3.26 (2.15,4.96)	3.17E-08	0.87	−0.259
rs59901167	15	48195306	0.968	intergenic	LINC01491	3.26 (2.14,4.96)	3.18E-08	0.87	−0.201
rs78689060	15	48194158	0.917	intergenic	LINC01491	3.26 (2.14,4.96)	3.23E-08	0.87	−0.22
rs112289874	15	48194686	0.917	intergenic	LINC01491	3.26 (2.14,4.96)	3.25E-08	0.87	−0.154
rs113194726	15	48195077	0.917	intergenic	LINC01491	3.26 (2.14,4.95)	3.25E-08	0.87	−0.286
rs16960326	15	48197491	0.972	intergenic	LINC01491	3.25 (2.14,4.95)	3.30E-08	0.87	−0.209
rs76540319	15	48151576	0.924	intergenic	LINC01491	3.36 (2.18,5.16)	3.36E-08	0.83	−0.141
rs147444776	15	48749199	0.973	intronic	FBN1	3.42 (2.21,5.30)	3.81E-08	0.76	−0.136
rs11857820	15	48204013	0.975	intergenic	LINC01491	3.22 (2.12,4.90)	4.02E-08	0.88	−0.167
rs148006967	15	48184958	0.958	intergenic	LINC01491	3.21 (2.11,4.87)	4.84E-08	0.89	−0.228

INFO denotes imputation score, OR (95% CI) denotes odds ratio with associated 95% interval, MAF denotes minor allele frequency, and eigenPC denotes Eigen PC score for genome-wide single nucleotide variants.

**TABLE 2:** ICD-10 diagnoses associated with PAH.

Diagnosis (ICD-10 code)	Cases (*n* = 493) (%)	Controls (*n* = 24,650) (%)	*p*-value
Tricuspid insufficiency (I07.1)	8	0	-
Atrial septal defect (Q21.1)	6	0	-
Dilated cardiomyopathy (I42.0)	6	0	-
Mitral (valve) prolapse (I34.1)	7	0	-
Pericardial effusion (non-inflammatory) (I31.3)	5	0	-
Congestive heart failure (I50.0)	29	1	0.0E+00
Pulmonary embolism without mention of acute cor pulmonale (I26.9)	12	1	2.0E-115
Peripheral vascular disease; unspecified (I73.9)	7	1	2.0E-40
Mitral (valve) insufficiency (I34.0)	25	1	0.0E+00
Chronic renal failure; unspecified (N18.9)	15	1	6.0E-170
Abnormal results of liver function studies (R94.5)	6	1	2.0E-24
Cardiomegaly (I51.7)	23	1	0.0E+00
Acute myocardial infarction; unspecified (I21.9)	6	1	2.0E-19
Presence of cardiac pacemaker (Z95.0)	14	1	6.0E-120
Pneumonia; unspecified (J18.9)	13	1	1.0E-99
Left ventricular failure (I50.1)	25	1	0.0E+00
Hypotension; unspecified (I95.9)	12	1	2.0E-92
Precordial pain (R07.2)	5	1	6.0E-15
Sleep apnea (G47.3)	5	1	8.0E-12
Pleural effusion; not elsewhere classified (J90)	20	1	1.0E-202
Hemorrhage and hematoma complicating a procedure; not elsewhere classified (T81.0)	9	2	3.0E-34
Arthrosis; unspecified (Site unspecified) (M19.99)	7	2	6.0E-18
Cellulitis of other parts of limb (L03.1)	8	2	3.0E-27
Acute renal failure; unspecified (N17.9)	23	2	5.0E-227
Anxiety disorder; unspecified (F41.9)	6	2	3.0E-14
Gastro-intestinal hemorrhage; unspecified (K92.2)	6	2	2.0E-11
Unstable angina (I20.0)	8	2	6.0E-22
Presence of aortocoronary bypass graft (Z95.1)	10	2	3.0E-39
Abnormal weight loss (R63.4)	6	2	8.0E-10
Lobar pneumonia; unspecified (J18.1)	18	2	7.0E-133
Hyperlipidemia; unspecified (E78.5)	5	2	2.0E-07
Dyspnea (R06.0)	24	2	1.0E-221
Iron deficiency anemia; unspecified (D50.9)	10	2	1.0E-30
Gastroenteritis and colitis of unspecified origin (A09.9)	6	2	3.0E-11
Headache (R51)	5	2	2.0E-06
Dysphagia (R13)	7	2	2.0E-13
Chronic obstructive pulmonary disease; unspecified (J44.9)	20	3	6.0E-101
Personal history of diseases of the nervous system and sense organs (Z86.6)	11	3	9.0E-23
Arthrosis; unspecified (M19.9)	9	3	5.0E-14
Nausea and vomiting (R11)	10	3	3.0E-16
Syncope and collapse (R55)	14	3	3.0E-41
Non-infective gastro-enteritis and colitis; unspecified (K52.9)	14	4	1.0E-25
Urinary tract infection; site not specified (N39.0)	16	4	3.0E-38
Family history of ischemic heart disease and other diseases of the circulatory system (Z82.4)	20	4	7.0E-64
Hypothyroidism; unspecified (E03.9)	13	5	6.0E-17
Chronic ischemic heart disease; unspecified (I25.9)	24	5	4.0E-84
Personal history of allergy to penicillin (Z88.0)	12	5	3.0E-11
Personal history of diseases of the circulatory system (Z86.7)	38	6	2.0E-182
Angina pectoris; unspecified (I20.9)	20	6	1.0E-37
Atrial fibrillation and flutter (I48)	53	6	0.0E+00
Non-insulin-dependent DM Without complications (E11.9)	21	6	2.0E-38
Chest pain; unspecified (R07.4)	25	7	2.0E-56
Atherosclerotic heart disease (I25.1)	30	7	5.0E-80
Asthma; unspecified (J45.9)	17	8	7.0E-14
Diaphragmatic hernia without obstruction or gangrene (K44.9)	17	10	7.0E-08
Personal history of psychoactive substance abuse (Z86.4)	38	11	2.0E-77
Pure hypercholesterolemia (E78.0)	38	13	1.0E-59
Essential (primary) hypertension (I10)	65	29	4.0E-64

**TABLE 3:** Biometrics and biomarkers in PAH cases and controls.

Biometric/Biomarker	Cases (n = 493)	Controls (n = 24,650)	*p*-value
BMI	28.7	27.5	7.8E-06
Height (in)	66.1	66.3	2.3E-01
Hip (in)	41.5	40.8	3.3E-04
Waist (in)	37.4	36.0	3.3E-07
Weight (lbs)	178.8	172.5	7.0E-04
Systolic Blood Pressure (mmHg)	141.5	144.4	4.7E-03
Diastolic Blood Pressure (mmHg)	80.6	82.5	1.1E-03
Mean Arterial Pressure (mmHg)	100.0	102.4	1.5E-04
Pulse Pressure (mmHg)	59.1	59.8	3.9E-01
Pulse Rate (bpm)	73.0	69.6	1.5E-06
Alkaline Phosphatase (U/L)	93.8	85.3	1.5E-06
Calcium (mg/dL)	9.5	9.5	1.7E-02
Vitamin D (ng/mL)	18.6	20.5	2.2E-06
C Reactive Protein (mg/dL)	130.0	77.2	1.1E-07
Apolipoprotein A (mg/dL)	148.0	155.0	3.0E-08
Apolipoprotein B (mg/dL)	95.1	104.0	2.0E-13
Total Cholesterol (mg/dL)	201.0	221.0	3.0E-18
HDL (mg/dL)	53.3	56.1	3.9E-04
LDL (mg/dL)	124.0	138.0	8.1E-17
Lipoprotein A (mg/dL)	19.8	21.0	3.3E-01
Triglycerides (mg/dL)	155.0	159.0	3.7E-01
Glucose (mg/dL)	97.3	93.3	1.5E-02
HbA1c (%)	5.7	5.5	5.9E-09
WBC (cells/mm3)	7670	6880	1.2E-10
Platelet Count (cells/uL)	249000	250000	8.1E-01
Hemoglobin (g/dL)	14.1	14.3	2.9E-03
Hematocrit (%)	41.0	41.3	1.3E-01
Mean Corpuscular Volume (fL)	92.0	91.6	4.2E-02
Albumin (g/dL)	4.4	4.5	4.7E-06
Blood Urea Nitrogen (mg/dL)	39.3	33.9	2.6E-12
Creatinine (mg/dL)	0.8	0.7	1.2E-04
CystatinC (mg/L)	1.1	0.9	6.8E-18
ALT (U/L)	22.1	23.5	7.3E-03
AST (U/L)	27.3	26.6	1.6E-01
GGT (U/L)	54.4	37.8	2.1E-06
Direct Bilirubin (mg/dL)	0.1	0.1	1.3E-07
Total Bilirubin (mg/dL)	0.6	0.6	1.1E-02
Total Protein (g/dL)	7.3	7.2	6.7E-02
Urate (mg/dL)	5.9	5.3	3.3E-13
Urine Microalbumin (mg/L)	68.8	28.2	3.7E-03

## Data Availability

The original contributions presented in the study are included in the article/Supplementary Material, further inquiries can be directed to the corresponding authors.
