# Supplementary material for: Identification of novel genetic variants, including PIM1 and LINC01491, with ICD-10 based diagnosis of pulmonary arterial hypertension in the UK Biobank cohort
Source: Front Drug Discov (Lausanne). Author manuscript; Available in PMC 2023 Apr 21. (PMC10121214; doi:10.3389/fddsv.2023.1127736)
Supplement: Data Sheet 1 [file NIHMS1892159-supplement-Data_Sheet_1.docx]

December 6, 2022

Dear Editors,

We are submitting with this letter a manuscript entitled “GWAS-based Evidence Linking PIM1 with Primary Arterial Hypertension” for consideration for publication as an Article in Frontiers in Drug Discovery, Hematological and Cardiovascular Drugs.

Here, using the UK Biobank cohort, we carried out a Genome-Wide Association Study (GWAS) of primary pulmonary hypertension (PPH) via ICD-10 diagnostic code. Our approach demonstrates the use of real-world data, such as the electronic health record, in combination with genetic information to gain new insights into rare diseases like PPH. Specifically, we show the first human genetic evidence linking PIM1,a protooncogene encoding a serine threonine kinase, to PAH. Our study provides further support for the growing body of literature suggesting PIM1 as a possible therapeutic target for PAH. Therefore, our findings may be of significant interest to your readers, particularly those interested in developing new therapies for PAH.

I attest that this paper is not under consideration elsewhere, that none of the paper’s contents have been previously published, that all authors have read and approved the manuscript, and that the full disclosure of any potential conflicts of interest have been made.

This study was conducted using the UK Biobank Resource under Application Number 49852 and received the proper ethical oversight, including the determination by the University of Maryland, Baltimore Institutional Review Board that the study is not human research (IRB #: HF-00088022).

We have listed requests for potential reviewers and their expertise attached.

Thank you for considering our manuscript.

Sincerely yours,


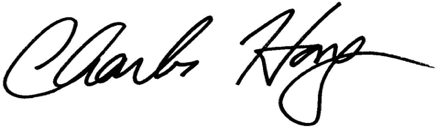


Charles C. Hong, MD, PhD

University of Maryland School of Medicine

Baltimore MD

Suggested reviewers:

Roxane Paulin, PhD, Université Laval, E-mail: roxane.paulin@criucpq.ulaval.ca

### Steeve Provencher, M.D., Université Laval. Email: steve.provencher@criucpq.ulaval.ca

Anna R. Hemnes, MD, Vanderbilt University School of Medicine. Email: anna.r.hemnes@vanderbilt.edu

James West, PhD, Vanderbilt University School of Medicine. Email: j.west@vumc.org

Rajeev Malhotra, MD, MGH/Harvard Medical School. Email: rmalhotra@mgh.harvard.edu

Hyung Chun, MD, Yale University. Email: hyung.chun@yale.edu
