# Supplementary material for: Identification of novel genetic variants, including PIM1 and LINC01491, with ICD-10 based diagnosis of pulmonary arterial hypertension in the UK Biobank cohort
Source: Front Drug Discov (Lausanne). Author manuscript; Available in PMC 2023 Apr 21. (PMC10121214; doi:10.3389/fddsv.2023.1127736)
Supplement: Data Sheet 3 [file NIHMS1892159-supplement-Data_Sheet_3.pdf]

Supplementary Material

GWAS-based Evidence Linking PIM1 with Primary Arterial Hypertension

Alex Pu[1], Yi-Ju Chen[1], Gautam Ramani, MD[1], James A. Perry, PhD[1*], Charles C. Hong, MD PhD[1*]

***Correspondence:**
Co-corresponding Authors
Charles C. Hong, MD, PhD: charles.hong@som.umaryland.edu
James A. Perry, PhD: jperry@som.umaryland.edu

1 Supplementary Data

Supplementary Material should be uploaded separately on submission. Please include any supplementary data, figures and/or tables.

Supplementary material is not typeset so please ensure that all information is clearly presented, the appropriate caption is included in the file and not in the manuscript, and that the style conforms to the rest of the article.

2 Supplementary Figures and Tables

For more information on Supplementary Material and for details on the different file types accepted, please see here.

2.1 Supplementary Figures

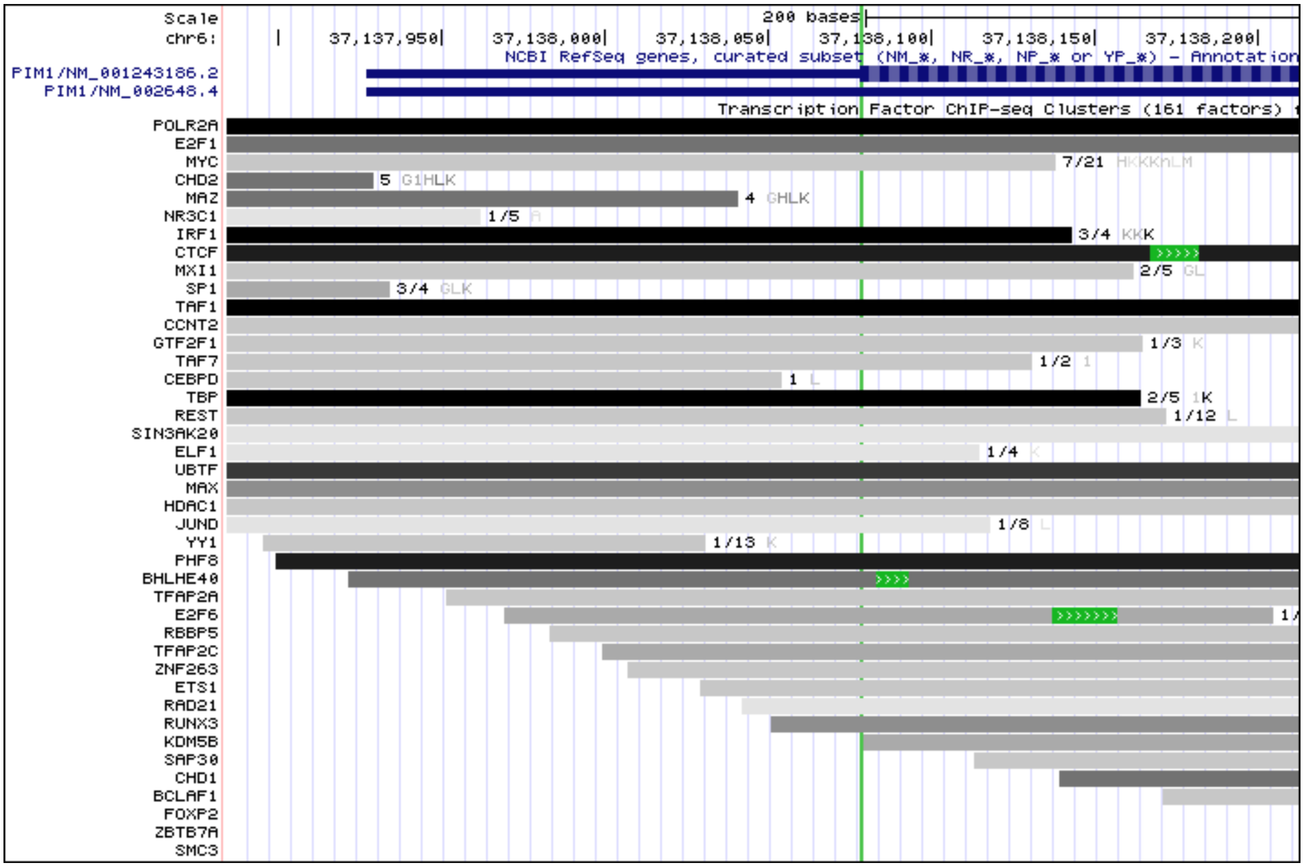

Supplementary Figure 1. UCSC Genome browser view of PIM1 genomic region. UCSC Genome Browser view of genomic region for PIM1 with view of intersecting transcription factor binding sites. Dark blue regions indicate PIM1 genomic region. Green vertical line denotes location of variant rs19244958. Gray regions enclose peak cluster of transcription factor occupancy from ENCODE/Duke. The darkness of each gray region is proportional to the maximum signal strength. The transcription factor name can be found to the left of each gray box.

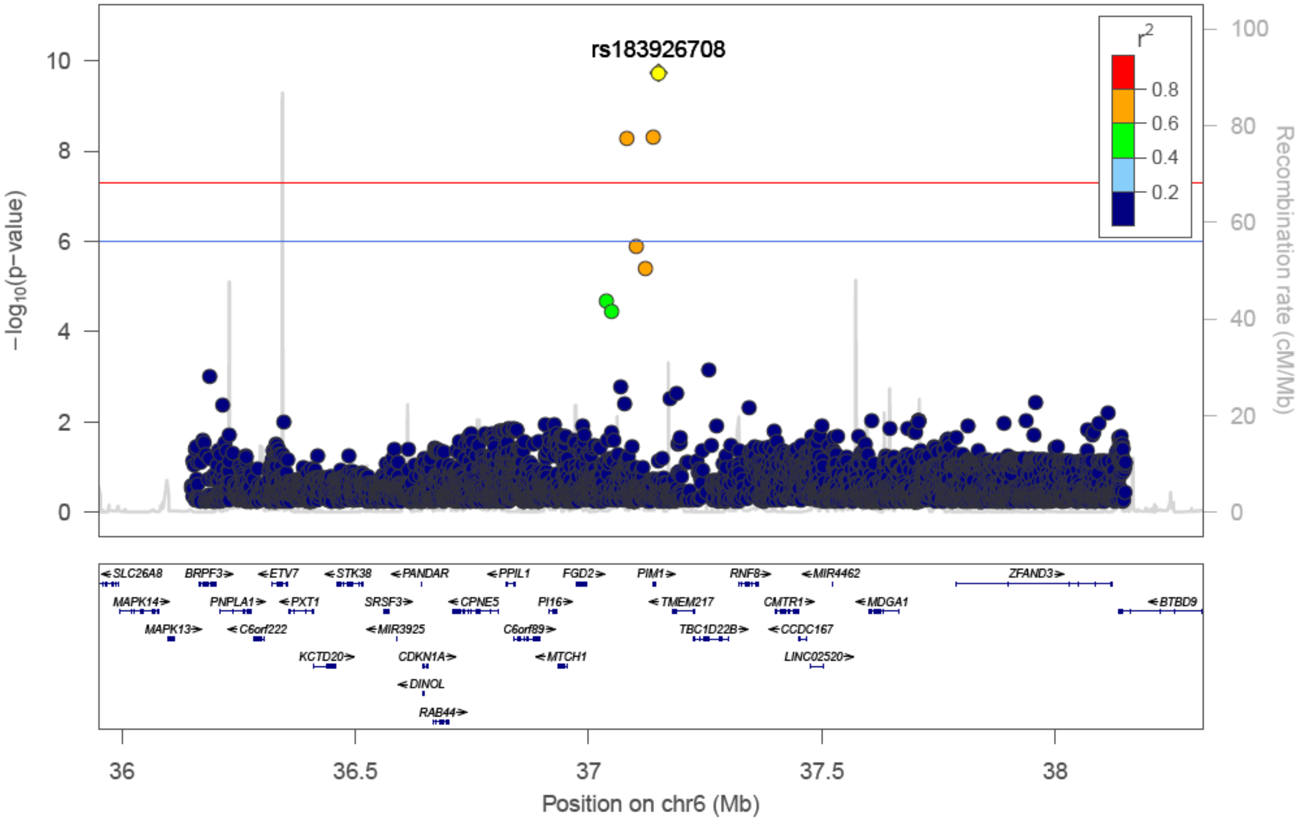

Supplementary Figure 2. Locus Zoom Plot of Top *PIM1* Variant. A zoomed in view of the Manhattan plot for the top variant rs183926708, with overlying genes underneath. Variants are colored according to their resulting r^2 value from linkage disequilibrium analysis, with rs183926708 (yellow) as the reference variant. The red line denotes genome wide significance at p = 5E-8. The significance is displayed on the y axis as -log10 of the p-value, and the results lie according to their location on chromosome 6 on the x axis. The recombination rate at different locations of chromosome 6 is delineated in gray as centiMorgan per megabase.

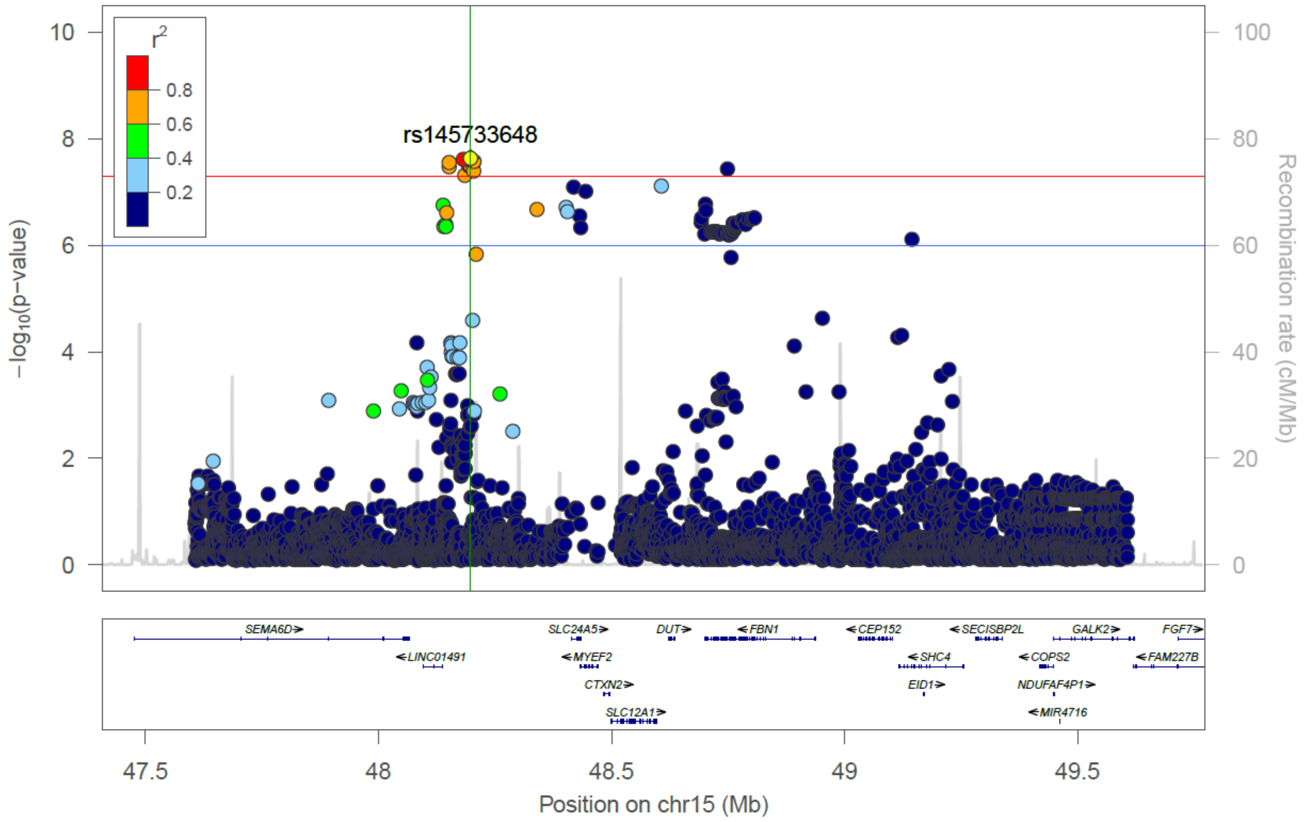

Supplementary Figure 3. Locus Zoom Plot of Top *LINC0149* Variant. A zoomed in view of the Manhattan plot for the top variant rs145733648, with overlying genes underneath. Variants are colored according to their resulting r^2 value from linkage disequilibrium analysis, with rs145733648 (yellow) as the reference variant. The red line denotes genome wide significance at p = 5E-8. The significance is displayed on the y axis as -log10 of the p-value, and the results lie according to their location on chromosome 15 on the x axis. The recombination rate at different locations of chromosome 15 is delineated in gray as centiMorgan per megabase.

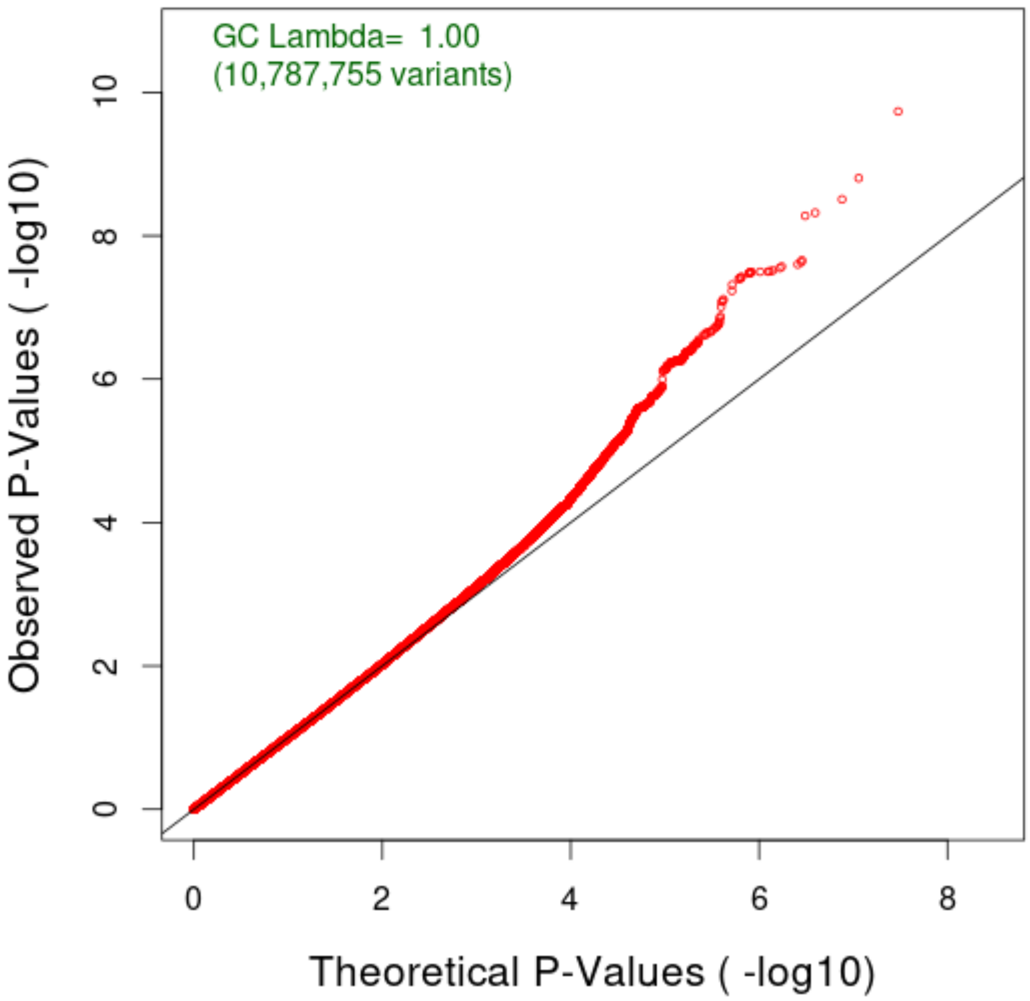

Supplementary Figure 4. Quantile-quantile plot (QQ Plot) for the Primary Pulmonary Hypertension phenotype showing the quality of the association analysis is well controlled with minimal confounding present. Red dots designate each variant. The -log10 of each variant's theoretical p-value is displayed on the x-axis, with the -log10 of the variant's observed p-value displayed on the y-axis. The genomic control (GC Lambda) was calculated to be 1.00, which is within the generally accepted range for GWAS.

2.2 Supplementary Tables

Supplementary Table 1. Regulatory motifs altered by variant rs192449585. Information regarding regulatory motifs affected by variant rs192449585 from HaploReg v4.1. For each affected regulatory motif, information regarding position weight matrix for the reference (Ref) and alternate (Alt) alleles are shown.

Position Weight Matrix ID	Strand	Ref	Alt
BCL_disc8	-	11.5	11.2
BCL_disc9	+	10.3	7.9
BHLHE40_disc2	-	11	8.3
ELF1_disc3	+	10.2	7.5
HEY1_disc2	-	12.2	10.4
NRSF_disc5	-	11.1	4.2
NRSF_disc8	-	11.3	5.2
Sin3Ak-20_disc6	-	10.3	-1.2
TATA_disc10	+	10.7	1.9
YY1_disc4	-	11.5	7.7
YY1_disc5	-	11.4	2.9
Znf143_disc4	+	10.6	8.8
p300_disc9	-	12.3	10.9

Supplementary Table 2. Genotype of Pulmonary Arterial Hypertension Cases and Controls. Breakdown of the number of cases and controls for each identified variant. HomRef denotes homozygous for the reference allele, Het denotes heterozygous for the alternative allele, HomAlt denotes homozygous for alternative allele.

		Cases			Control		
Variant	Gene	HomRef	Het	HomAlt	HomRef	Het	HomAlt
rs567757955	LOC101927237	475	18	0	24396	253	1
rs187386578	PIM1	470	23	0	24303	345	2
rs192449585	PIM1	469	24	0	24306	342	2
rs183926708	PIM1	468	25	0	24309	339	2
rs370775256	LOC101927003	459	34	0	24023	620	7
rs573886591	CSMD3	463	30	0	24135	513	2
rs4764961	C12orf42	428	63	2	23005	1620	25
rs193148583	KIF26A	473	20	0	24338	312	0
rs76540319	LINC01491	470	23	0	24268	382	0
rs80073095	LINC01491	470	23	0	24267	383	0
rs60777293	LINC01491	469	24	0	24254	396	0
rs148006967	LINC01491	469	24	0	24235	415	0
rs78689060	LINC01491	469	24	0	24244	406	0
rs113347288	LINC01491	469	24	0	24244	406	0
rs112289874	LINC01491	469	24	0	24244	406	0
rs113194726	LINC01491	469	24	0	24244	406	0
rs59901167	LINC01491	469	24	0	24244	406	0
rs61518006	LINC01491	469	24	0	24244	406	0
rs145733648	LINC01491	469	24	0	24252	398	0
rs16960326	LINC01491	469	24	0	24244	406	0
rs77487976	LINC01491	469	24	0	24246	404	0
rs11857820	LINC01491	469	24	0	24241	409	0
rs113318990	LINC01491	469	24	0	24248	402	0
rs147444776	FBN1	470	23	0	24285	365	0
