# Supplementary material for: Identification of novel genetic variants, including PIM1 and LINC01491, with ICD-10 based diagnosis of pulmonary arterial hypertension in the UK Biobank cohort
Source: Front Drug Discov (Lausanne). Author manuscript; Available in PMC 2023 Apr 21. (PMC10121214; doi:10.3389/fddsv.2023.1127736)
Supplement: Data Sheet 2 [file NIHMS1892159-supplement-Data_Sheet_2.docx]

Supplementary Material

GWAS-based Evidence Linking PIM1 with Primary Arterial Hypertension

Alex Pu^1^, Yi-Ju Chen^1^, Gautam Ramani, MD^1^, James A. Perry, PhD^1*^, Charles C. Hong, MD PhD^1*^

***Correspondence:**Co-corresponding Authors
Charles C. Hong, MD, PhD: [charles.hong@som.umaryland.edu](mailto:charles.hong@som.umaryland.edu)
James A. Perry, PhD: [jperry@som.umaryland.edu](mailto:jperry@som.umaryland.edu)

# Supplementary Data

Supplementary Material should be uploaded separately on submission. Please include any supplementary data, figures and/or tables.

Supplementary material is not typeset so please ensure that all information is clearly presented, the appropriate caption is included in the file and not in the manuscript, and that the style conforms to the rest of the article.

# Supplementary Figures and Tables

For more information on Supplementary Material and for details on the different file types accepted, please see [here](https://www.frontiersin.org/guidelines/author-guidelines#supplementary-material).

## Supplementary Figures


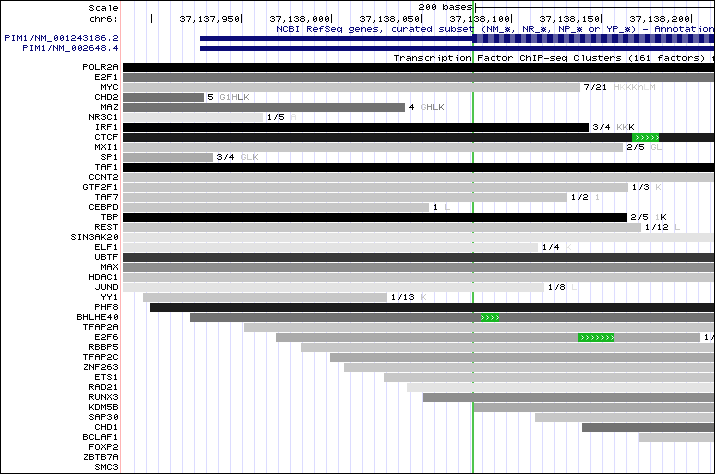


**Supplementary Figure 1.** **UCSC Genome browser view of PIM1 genomic region.** UCSC Genome Browser view of genomic region for PIM1 with view of intersecting transcription factor binding sites. Dark blue regions indicate PIM1 genomic region. Green vertical line denotes location of variant rs19244958. Gray regions enclose peak cluster of transcription factor occupancy from ENCODE/Duke. The darkness of each gray region is proportional to the maximum signal strength. The transcription factor name can be found to the left of each gray box.


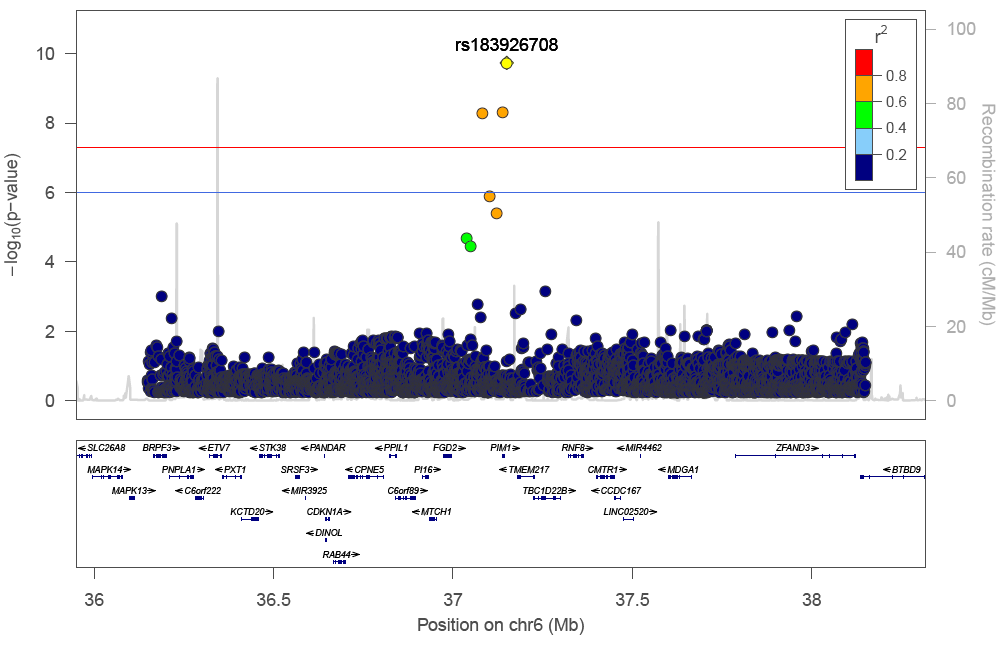


**Supplementary Figure 2.** **Locus Zoom Plot of Top *PIM1* Variant.** A zoomed in view of the Manhattan plot for the top variant rs183926708, with overlying genes underneath. Variants are colored according to their resulting r^2^ value from linkage disequilibrium analysis, with rs183926708 (yellow) as the reference variant. The red line denotes genome wide significance at p = 5E-8. The significance is displayed on the y axis as -log10 of the p-value, and the results lie according to their location on chromosome 6 on the x axis. The recombination rate at different locations of chromosome 6 is delineated in gray as centiMorgan per megabase.

**
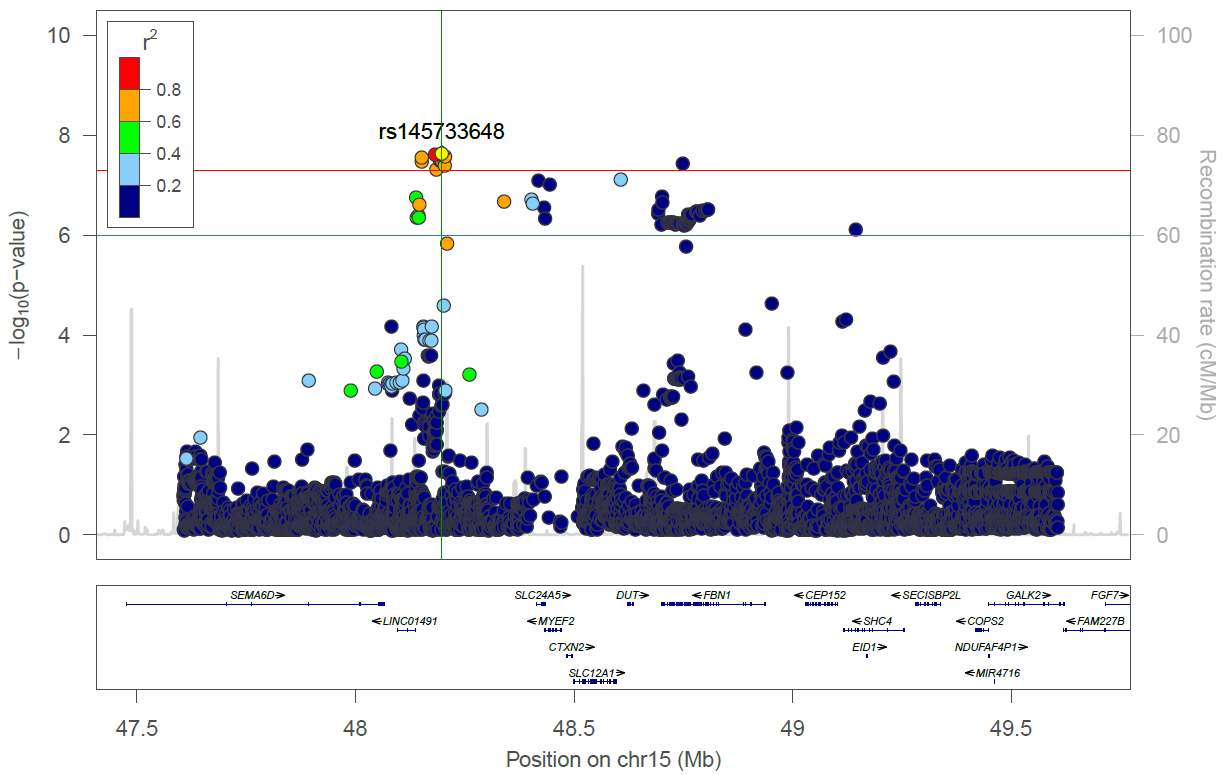
**

**Supplementary Figure 3.** **Locus Zoom Plot of Top *LINC0149* Variant.** A zoomed in view of the Manhattan plot for the top variant rs145733648, with overlying genes underneath. Variants are colored according to their resulting r^2^ value from linkage disequilibrium analysis, with rs145733648 (yellow) as the reference variant. The red line denotes genome wide significance at p = 5E-8. The significance is displayed on the y axis as -log10 of the p-value, and the results lie according to their location on chromosome 15 on the x axis. The recombination rate at different locations of chromosome 15 is delineated in gray as centiMorgan per megabase.

**
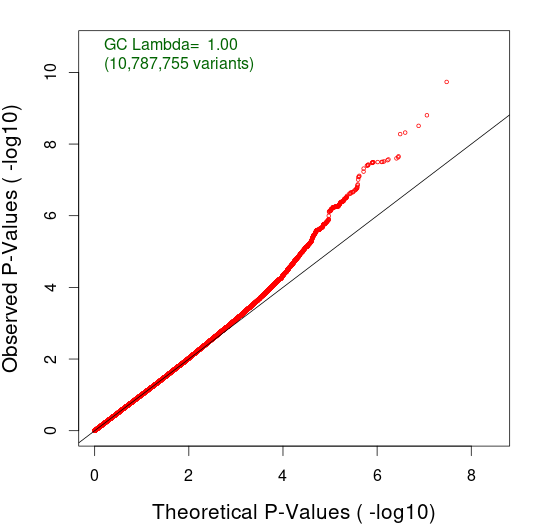
**

**Supplementary Figure 4.** **Quantile-quantile plot (QQ Plot) for the Primary Pulmonary Hypertension phenotype showing the quality of the association analysis is well controlled with minimal confounding present.** Red dots designate each variant. The -log10 of each variant’s theoretical p-value is displayed on the x-axis, with the -log10 of the variant’s observed p-value displayed on the y-axis. The genomic control (GC Lambda) was calculated to be 1.00, which is within the generally accepted range for GWAS.

## Supplementary Tables

**Supplementary Table 1. Regulatory motifs altered by variant rs192449585.** Information regarding regulatory motifs affected by variant rs192449585 from HaploReg v4.1. For each affected regulatory motif, information regarding position weight matrix for the reference (Ref) and alternate (Alt) alleles are shown.

| **Position Weight Matrix ID** | **Strand** | **Ref** | **Alt** |
| --- | --- | --- | --- |
| BCL_disc8 | - | 11.5 | 11.2 |
| BCL_disc9 | + | 10.3 | 7.9 |
| BHLHE40_disc2 | - | 11 | 8.3 |
| ELF1_disc3 | + | 10.2 | 7.5 |
| HEY1_disc2 | - | 12.2 | 10.4 |
| NRSF_disc5 | - | 11.1 | 4.2 |
| NRSF_disc8 | - | 11.3 | 5.2 |
| Sin3Ak-20_disc6 | - | 10.3 | -1.2 |
| TATA_disc10 | + | 10.7 | 1.9 |
| YY1_disc4 | - | 11.5 | 7.7 |
| YY1_disc5 | - | 11.4 | 2.9 |
| Znf143_disc4 | + | 10.6 | 8.8 |
| p300_disc9 | - | 12.3 | 10.9 |

**Supplementary Table 2. Genotype of Pulmonary Arterial Hypertension Cases and Controls.** Breakdown of the number of cases and controls for each identified variant. HomRef denotes homozygous for the reference allele, Het denotes heterozygous for the alternative allele, HomAlt denotes homozygous for alternative allele.

|  |  | Cases | | | Control | | |
| --- | --- | --- | --- | --- | --- | --- | --- |
| Variant | Gene | HomRef | Het | HomAlt | HomRef | Het | HomAlt |
| rs567757955 | LOC101927237 | 475 | 18 | 0 | 24396 | 253 | 1 |
| rs187386578 | PIM1 | 470 | 23 | 0 | 24303 | 345 | 2 |
| rs192449585 | PIM1 | 469 | 24 | 0 | 24306 | 342 | 2 |
| rs183926708 | PIM1 | 468 | 25 | 0 | 24309 | 339 | 2 |
| rs370775256 | LOC101927003 | 459 | 34 | 0 | 24023 | 620 | 7 |
| rs573886591 | CSMD3 | 463 | 30 | 0 | 24135 | 513 | 2 |
| rs4764961 | C12orf42 | 428 | 63 | 2 | 23005 | 1620 | 25 |
| rs193148583 | KIF26A | 473 | 20 | 0 | 24338 | 312 | 0 |
| rs76540319 | LINC01491 | 470 | 23 | 0 | 24268 | 382 | 0 |
| rs80073095 | LINC01491 | 470 | 23 | 0 | 24267 | 383 | 0 |
| rs60777293 | LINC01491 | 469 | 24 | 0 | 24254 | 396 | 0 |
| rs148006967 | LINC01491 | 469 | 24 | 0 | 24235 | 415 | 0 |
| rs78689060 | LINC01491 | 469 | 24 | 0 | 24244 | 406 | 0 |
| rs113347288 | LINC01491 | 469 | 24 | 0 | 24244 | 406 | 0 |
| rs112289874 | LINC01491 | 469 | 24 | 0 | 24244 | 406 | 0 |
| rs113194726 | LINC01491 | 469 | 24 | 0 | 24244 | 406 | 0 |
| rs59901167 | LINC01491 | 469 | 24 | 0 | 24244 | 406 | 0 |
| rs61518006 | LINC01491 | 469 | 24 | 0 | 24244 | 406 | 0 |
| rs145733648 | LINC01491 | 469 | 24 | 0 | 24252 | 398 | 0 |
| rs16960326 | LINC01491 | 469 | 24 | 0 | 24244 | 406 | 0 |
| rs77487976 | LINC01491 | 469 | 24 | 0 | 24246 | 404 | 0 |
| rs11857820 | LINC01491 | 469 | 24 | 0 | 24241 | 409 | 0 |
| rs113318990 | LINC01491 | 469 | 24 | 0 | 24248 | 402 | 0 |
| rs147444776 | FBN1 | 470 | 23 | 0 | 24285 | 365 | 0 |
